# Complications during pharmacological stress echocardiography: a video-case series

**DOI:** 10.1186/1476-7120-3-25

**Published:** 2005-09-02

**Authors:** Albert Varga, Giuliano Kraft, Ferenc Lakatos, Riccardo Bigi, Rafael Paya, Eugenio Picano

**Affiliations:** 1 2nd Department of Medicine, University of Sciences, Szeged, Hungary; 2 Institute of Clinical Physiology, Pisa, Italy; 3 Town Hospital, Orosháza, Hungary; 4 Cardiology, Department of Medicine and Surgery, University School of Medicine, S. Paolo Academic Hospital, Milan, Italy; 5 Research Center La Fe Hospital, Valencia, Spain

## Abstract

**Background:**

Stress echocardiography is a cost-effective tool for the modern noninvasive diagnosis of coronary artery disease. Several physical and pharmacological stresses are used in combination with echocardiographic imaging, usually exercise, dobutamine and dipyridamole. The safety of a stress is (or should be) a major determinant in the choice of testing. Although large scale single center experiences and multicenter trial information are available for both dobutamine and dipyridamole stress echo testing, complications or side effects still can occur even in the most experienced laboratories with the most skilled operators.

**Case presentation:**

We decided to present a case collection of severe complications during pharmacological stress echo testing, including a ventricular tachycardia, cardiogenic shock, transient ischemic attack, torsade de pointe, fatal ventricular fibrillation, and free wall rupture.

**Conclusion:**

We believe that, in this field, every past complication described is a future complication avoided; what happens in your lab is more true of what you read in journals; and Good Clinical Practice is not "not having complications", but to describe the complications you had.

## Background

The safety of the stress test is a major issue in deciding its practicability and cost-effectiveness – yet, many major complications remain "unmentioned and unheard", for several reasons – mainly related to the "file drawer" bias, lack of time ("busy agenda bias") or unfamiliarity with the technicalities of scientific communication (editorial "black box bias").

Stress echocardiography is a cost-effective tool for the modern noninvasive diagnosis of coronary artery disease [1]. Several physical and pharmacological stresses are used in combination with echocardiographic imaging, usually exercise, dobutamine and dipyridamole. The safety of a stress is (or should be) a major determinant in the choice of testing. Although large scale single center experiences and multicenter trial information are available for both dobutamine and dipyridamole [2-6] stress echo testing, complications or side effects still can occur even in the most experienced laboratories with the most skilled operators. We believe that, in this field, every past complication described is a future complication avoided; what happens in your lab is more true than what you read in journals; and Good Clinical Practice is not "not having complications", but to describe the complications you had. Therefore, we decided to present an unusual case series, consisting in a collection of severe complications during pharmacological stress echo testing.

## Case presentation

### Case 1

A 73 year-old male patient, with a previous PTCA (percutaneous transluminal coronary angioplasty) of the left anterior descending artery and ramus intermedius, underwent a dipyridamole stress testing following a nondiagnostic exercise EKG (the exercise was terminated because of the occurrence of non sustained ventricular tachycardia). The baseline echo revealed an apical hypokinesis (additional file 1) which did not change during the test, however ventricular tachycardia developed again during dipyridamole echo (additional file 2). Lesson: it is useless to expose a patient with known coronary artery disease and a previously complicated test to another stressor. Indication must be appropriate.

### Case 2

An 81 year-old female, with symptomatic and hemodynamically significant aortic stenosis and normal coronary angiogram underwent a high dose dipyridamole stress echo testing. The baseline wall motion was normal (additional file 3). The patient fell in cardiogen shock and had a transient ischemic attack of the brain following a negative test (additional file 4). Lesson: another dangerous experiment on a patient with already diagnosed normal coronary arteries. Indication must be always appropriate.

### Case 3

A 57 year-old male patient with abdominal pain and claudicatio intermittens was studied with dobutamine echocardiography. Soon after the first (5 mcg/Kg/min) dose the patient had ventricular extrasystoles (additional file 5) and during the 20 mcg/Kg/min dose of dobutamine, Torsade de points ventricular tachycardia evolved (additional file 6). Lesson: in patients with arrhytmias in resting conditions, dobutamine can often provoke dangerous tachycardias. In this group of patients dipyridamole could be the first choice.

### Case 4

A 55 year-old male patient with previous posterior myocardial infarction, quadruple by-pass, depressed left ventricular function and chest pain was sent to the echo lab for assessment of myocardial viability (additional file 7). Low dose dobutamine echo was performed, however, following the 10 mcg/Kg/min dose a fatal ventricular fibrillation developed (additional file 8). Lesson: there must always be an attending physician during pharmacological stress echo testing with all necessary equipment for reanimation. Dobutamine can provoke arrhytmias even in low doses.

### Case 5

A 66 year-old male patient with a recent (12 days old) inferior infarction and inferior aneurysm underwent a high dose dobutamine stress test. A huge aneurysm of the inferior wall was present on the baseline echocardiogram (additional file 9). The patient died following an acute cardiac rupture (additional file 10). Lesson: indications for testing must always be first class, and in patients with recent infarction and aneurysm dipyridamole should be the first choice.

## Conclusion

As stated in the American College of Cardiology/American Heart Association Clinical Competence Statement on Stress Testing, cognitive skills are required to attain competence in the direct supervision of stress echocardiographic tests, but not only the knowledge of the complications of different pharmacological agents but also the knowledge of their complication rate is important [7]. Therefore, both the patient and the physician, should be fully aware of the rate of complications during the application of all forms of stress. It is our stress policy, in the everyday echo lab activity, to strictly adopt the following criteria based on conventional wisdom and evidence-based medicine: 1) Avoid contraindications; 2) Never exceed standard dosages; 3) Perform the test after signed information consent has been obtained; 4) There must always be an attending physician; 5) Outpatients should be kept for 60' in the waiting room after testing; 6) Indications for testing must be class first class.

## Supplementary Material

Additional File 1 The baseline echo (apical 4 chamber view) with apical hypokinesis. Click here for file

Additional File 2 No change in wall motion, but ventricular tachycardia developed at peak stress. Click here for file

Additional File 3 Resting parasternal short axis view and apical 4 chamber view with normal regional left ventricular wall motion. Click here for file

Additional File 4 Following the dipyridamole administration cardiogenic shock occurred. Depressed global left ventricular function can be seen both from parasternal long axis view and apical 4 chamber view. Click here for file

Additional File 5 Apical 4 chamber view during low dose dobutamine. Click here for file

Additional File 6 Parasternal long axis view. The initiation of the torsade de pointe ventricular tachycardia. Click here for file

Additional File 7 Apical 4 chamber view and apical long axis view. Apical and posterior akinesia on the resting images. Click here for file

Additional File 8 Ventricular fibrillation following a low dose dobutamine. Click here for file

Additional File 9 Quad-screen image of a patient with inferior aneurysm. Click here for file

Additional File 10 The image of the heart following a cardiac rupture with huge pericardial effusion. Click here for file
